# The Effects of Glutamine Supplementation on Liver Inflammatory Response and Protein Metabolism in Muscle of Lipopolysaccharide-Challenged Broilers

**DOI:** 10.3390/ani14030480

**Published:** 2024-02-01

**Authors:** Bolin Zhang, Qian Yang, Ning Liu, Qingzhen Zhong, Zewei Sun

**Affiliations:** 1 College of Animal Science and Technology, Qingdao Agricultural University, Chang Cheng Road, Cheng Yang District, Qingdao 266109, China; 2 Department of Biology and Agriculture, Zunyi Normal College, Ping’an Avenue, Hong Huagang District, Zunyi 563006, China; 3 College of Animal Science and Technology, Jilin Agricultural University, No. 2888, Xincheng Road, Jingyue District, Changchun 130118, China; yq1235@126.com (Q.Y.); lxbolin@163.com (N.L.); qingzhenzhong@163.com (Q.Z.)

**Keywords:** lipopolysaccharide challenge, glutamine, liver inflammation, protein synthesis, protein degradation

## Abstract

**Simple Summary:**

Our previous study suggested that glutamine (Gln), defined as a conditionally essential amino acid, contributed to improving growth performance, alleviating inflammatory responses and intestinal permeability, as well as rescuing the destroyed intestinal mucosa induced by LPS exposure. In addition, it was demonstrated that LPS-induced immune stress led to a severe loss of muscle mass. Here, Gln was proven to function in regulating inflammatory responses, protein synthesis, and degradation during sepsis. Our results showed that Gln administration attenuated liver inflammatory reactions, elevated protein synthesis, and inhibited protein degradation of broilers subjected to LPS challenge.

**Abstract:**

The aim of our present study was to investigate the effects of Gln supplementation on liver inflammatory responses as well as protein synthesis and degradation in the muscle of LPS-challenged broilers. A total of 120 one-day-old male broiler chickens (Arbor Acres Plus) were randomly arranged in a 2 × 2 factorial design with five replicates per treatment and six broilers per replicate, containing two main factors: immune challenge (injected with LPS in a dose of 0 or 500 µg/kg of body weight) and dietary treatments (supplemented with 1.22% alanine or 1% Gln). After feeding with an alanine or Gln diet for 15 days, broilers were administrated an LPS or a saline injection at 16 and 21 days. The results showed that Gln supplementation alleviated the increased mRNA expressions of *interleukin-6*, *interleukin-1β*, and *tumor necrosis factor-α* induced by LPS in liver. Moreover, the increased activity of aspartate aminotransferase combined with the decreased expression of glutaminase in muscle were observed following Gln addition. In addition, in comparison with the saline treatment, LPS challenge altered the signaling molecules’ mRNA expressions associated with protein synthesis and degradation. However, Gln supplementation reversed the negative effects on protein synthesis and degradation in muscle of LPS-challenged broilers. Taken together, Gln supplementation had beneficial effects: alleviating inflammatory responses, promoting protein synthesis, and inhibiting protein degradation of LPS-challenged broilers.

## 1. Introduction

Under intensive breeding conditions, broilers commonly suffer from immune stress that is induced by diverse pathogenic and nonpathogenic microorganisms, which cause adverse changes in body metabolism and immune system and ultimately result in impaired growth performance [1]. Lipopolysaccharide (LPS), an active component of Gram-negative bacterial cell membranes and an effective immunostimulatory component, can be preferentially used to establish an immune stress model via intraperitoneal injection in broilers [2,3]. Previous studies proved that an acute systemic inflammatory response, such as the production of inflammatory factors and the altered expression of genes involved in the immune system, occur in response to LPS stimulation [4]. Our previous study showed that LPS challenge resulted in impaired growth performance, elevated inflammatory cytokines in serum, and destroyed intestinal mucosal barriers in broilers [5]. In a previous study, it was also demonstrated that LPS-induced immune stress led to a severe loss of muscle mass [6]. Moreover, LPS challenge altered the molecular pathways regulating the catabolism of muscle mass, which contain the signaling molecules responsible for increased protein degradation and decreased protein synthesis [7].

It has been well documented that the rapamycin mammalian target of rapamycin (mTOR) signaling pathway is responsible for protein synthesis in skeletal muscle and that the activation of the ubiquitin–proteasome pathway primarily mediates the protein degradation of skeletal muscle [7,8]. It was reported that LPS administration depressed protein synthesis in skeletal muscle, associated with the reduced expression of eukaryotic initiation factor eIF4E binding protein 1 (4E-BP1) and ribosomal protein 6 kinase 1 (S6K1), which are the downstream targets of the mTOR signaling pathway that is involved in protein synthesis [9,10]. In addition, LPS challenge resulted in increased expressions of muscle atrophy F-box (*MAFbx*) and muscle ring finger 1 (*MuRF1*), which are considered the key regulators of muscle protein degradation [7,11]. Therefore, it is worthwhile to search for effective measures of nutritional modulation to suppress proinflammatory cytokines and attenuate muscle atrophy in broilers during sepsis and inflammation.

The concentration of glutamine (Gln), which is the most abundant amino acid in plasma and skeletal muscle, falls dramatically in the circulation and tissues during stress conditions [12]. Therefore, Gln is a conditionally essential amino acid during inflammatory conditions [13]. It has been demonstrated that Gln has a wide range of biological functions and serves important roles in promoting growth performance, maintaining intestinal health, and improving immune responses [5,14,15,16]. Dai et al. [12] suggested that dietary supplementation with 1% Gln improved the growth performance of broilers suffering from heat stress. Xue et al. [17] demonstrated that compared with broilers subjected to necrotic enteritis challenge, 1% Gln supplementation increased their feed intake and weight gain and decreased the feed conversion ratio. Furthermore, Gln addition contributed to alleviating the inflammatory reactions evidenced as decreased concentrations of tumor necrosis factor-α (TNF-α), interleukin-1β (IL-1β), and interleukin-6 (IL-6) in the plasma [5]. Additionally, Gln is considered to be one of the important amino acids in the regulation of the mTOR pathway and autophagy [18,19], which are responsible for protein synthesis and degradation, respectively. It has been demonstrated that the in vitro addition of Gln elevated the protein synthesis and reduced the protein degradation of porcine intestinal epithelial cells [20].

However, the effects of Gln regulation on the protein synthesis and degradation of muscle in LPS-challenged broilers is still not well understood. Therefore, the objective of the current study was to investigate the effects of Gln supplementation on liver inflammatory responses and the protein synthesis and degradation in the muscles of LPS-challenged broilers.

## 2. Materials and Methods

### 2.1. Diets, Experimental Design, and Animal Management

The protocols of this experiment were performed according to the guidelines of the Institutional Animal Care and Use Committee of Zunyi Normal College and approved by the Animal Care and Use Committee.

The dosages of Gln (1.0%) supplementation and Ala (1.22%) as the isonitrogenous control were set according to our previous study [5]. Both Ala and Gln were purchased from Shanghai Feiya Technology Development Co., Ltd. (Shanghai, China).

One hundred and twenty one-day-old male broilers (Arbor Acres Plus) were randomly arranged in a 2 × 2 factorial design, containing five replicates per treatment and six broilers per replicate. Broilers were fed either an Ala or a Gln diet for 21 days. After 15 days of feeding broilers were intraperitoneally injected with LPS solution at a dosage of 500 μg LPS/kg of body weight or an equal volume of 0.9% sterile saline at 8.00 a.m. on days 16 and 21, respectively. The four experimental treatments were as follows: (1) Ala-saline group, in which birds were fed a diet containing 1.22% Ala and received intraperitoneal administration of sterile saline; (2) Gln-saline group, in which birds were fed a diet containing 1% Gln and received intraperitoneal administration of sterile saline; (3) Ala-LPS group, in which birds were fed a diet containing 1.22% Ala and received intraperitoneal LPS injection; (4) Gln-LPS group, in which birds were fed a diet containing 1% Gln and received intraperitoneal LPS injection. All broilers were kept in a temperature-controlled room with a humidity of 60–65% and a light protocol of 23 h light and 1 h dark. The temperature was kept at 34–36 °C and gradually decreased by 2–3 °C weekly until it reached a final temperature of around 26 °C. The growth performance of broilers receiving the aforementioned treatments was reported in our previous study [5]. The broilers used in the present study were purchased from Jilin Dexiang Animal Husbandry Co., Ltd. (Changchun, China). The ingredients of the diets and nutrition contents are presented in Table 1. Throughout the entire experiment, broilers were given ad libitum access to feed in mash form and fresh water.

### 2.2. Sample Collection

Two hours after LPS injection, 10 broilers at 21 days of age (2 broilers per replicate) were randomly selected to obtain blood samples from the wing vein. The collected blood samples were centrifuged at 4 °C and 3000× *g* for 10 min to collect plasma and stored at −20 °C until further analysis. Immediately after blood sampling, the selected broilers were sacrificed via cervical dislocation followed by exsanguination. About 10 g *Pectoralis Major* muscle was collected into sterile tubes and stored in liquid nitrogen until subsequent analysis. All samples were collected within 10 min of euthanasia.

### 2.3. Determination of Enzyme Activities in Pectoralis Major Muscles

The activities of alanine aminotransferase (ALT), aspartate aminotransferase (AST), and glutamine synthetase (GS) in *Pectoralis Major* muscles were determined using commercial kits purchased from Nanjing Jiancheng Bioengineering Institute (Nanjing, China). The protocols were conducted according to the instructions of the manufacturers.

### 2.4. Real-Time PCR Analysis

Total RNA extractions from *Pectoralis Major* muscle samples using RNAiso Plus reagent (catalogue No. 9108, TaKaRa Biotechnology (Dalian) Co., Ltd., Dalian China) were reverse-transcribed into cDNA using PrimeScript TM RT Master Mix (catalogue No. RR037A, TaKaRa), followed by real-time RT-PCR analysis with TB Green Premix Ex Taq (catalogue No. RR420A), according to the instructions of the manufacturer. The PCR program consisted of one cycle at 95 °C for 30 s, 40 cycles of denaturation at 95 °C for 5 s, followed by a 60 °C annealing step for 30 s. The expressions of selected genes relative to the housekeeping gene (β-actin) were analyzed according to the method of Livak and Schmittgen [21]. The primer sequences of the target genes are shown in Table 2: toll-like receptor 4 (*TLR4*), forkhead box O1 (*FOXO1*), forkhead box O4 (*FOXO4*), protein kinase B (*Akt*), *MAFbx*, *MuRF1*, *mTOR*, *4E-BP1*, *S6K1*, and *β-actin*.

### 2.5. Statistical Analysis

Data analysis was conducted using a 2 × 2 factorial arrangement (SPSS version 20.0), and significant differences among different treatments were analyzed using two-factorial analysis of variance. The statistical model contained diet (1.2% Ala or 1.0% Gln) and intraperitoneal injection challenge (LPS or saline) and their interactions. *p* < 0.05 was considered significant. All values are shown as means and standard error of the mean.

## 3. Results

### 3.1. mRNA Expressions of TNF-α, IL-6, IL-1β in the Liver

As illustrated in Table 3, there were significant interactions for liver IL-6 and IL-1β mRNA expression between Gln addition and LPS challenge (*p* < 0.05). But, no interaction for *TNF-α* mRNA expression in liver was observed between Gln addition and LPS challenge (*p* > 0.05) In comparison with those receiving saline treatment, LPS challenge significantly increased the mRNA expression of *TNF-α* in the livers of the broilers (*p* < 0.05). Similarly, the *IL-6* and *IL-1β* mRNA expressions in the livers of the broilers were also elevated by LPS challenge (*p* < 0.05). However, Gln supplementation decreased the increased expression of *TNF-α* in the liver. Moreover, Gln addition reversed the mRNA expressions of *IL-6* and *IL-1β* in the livers of the broilers that were stimulated via LPS challenge (*p* < 0.05).

### 3.2. The Activities of ALT and AST in the Muscle of Broilers

As shown in Table 4, no interaction for the activities of AST or ALT was observed between the LPS treatment and Gln supplementation (*p* > 0.05). The activity of AST in the *Pectoralis Major* muscle of the broilers decreased with LPS administration in comparison with those treated with saline injection (*p* < 0.05). However, no difference in the activity of ALT in the *Pectoralis Major* muscle of the broilers was observed in the LPS treatment group (*p* > 0.05). In contrast, compared with the broilers fed an Ala-supplemented diet, dietary Gln supplementation significantly elevated the activity of AST in the *Pectoralis Major* muscle of the broilers (*p* < 0.05). But, Gln supplementation did not affect the activity of ALT in the *Pectoralis Major* muscle of the broilers (*p* > 0.05).

### 3.3. GS Activity and mRNA Expression of GA in the Pectoralis Major Muscle of Broilers

**Figure 1:**
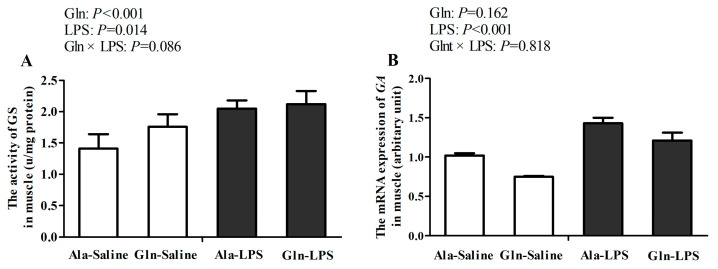
Effects of Gln supplementation on GS activity (**A**) and *GA* mRNA expression (**B**) in muscle of 21-day-old LPS-challenged broilers. GS, glutamine synthetase; GA, glutaminase; Ala, alanine; Gln, glutamine; LPS, lipopolysaccharide. Values are means ± SEM, *n* = 10.

As presented in Figure 1, there were no interactions for *GA* mRNA expression between LPS challenge and Gln supplementation (*p* > 0.05). However, a significant trend was observed in the activity of GS between LPS challenge and Gln supplementation (*p* = 0.086). In comparison with the broilers receiving saline injection, LPS challenge significantly increased the activity of GS in the *Pectoralis Major* muscle of the broilers (*p* < 0.05). Meanwhile, the mRNA expressions of *GA* in the *Pectoralis Major* muscle of LPS-challenged broilers were also upregulated by LPS treatment (*p* < 0.05). However, Gln supplementation increased the activity of GS in the *Pectoralis Major* muscle of the broilers in comparison with those receiving diets supplemented with Ala (*p* < 0.05). But, the increased mRNA expression of *GA* was not affected by Gln supplementation (*p* > 0.05).

### 3.4. mTOR Signaling Molecules in the Pectoralis Major Muscle

**Figure 2:**
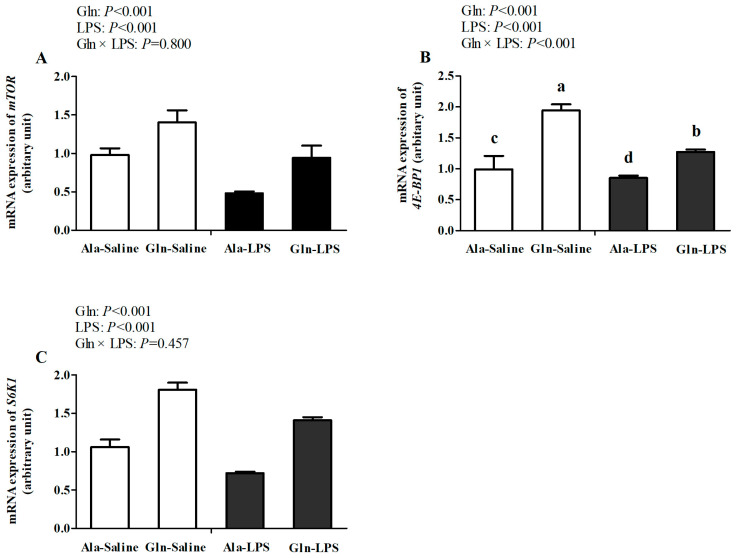
Effects of Gln supplementation on mRNA expressions of *mTOR* (**A**), *4E-BP1* (**B**), and *S6K1* (**C**) in muscle of 21-day-old LPS-challenged broilers. *mTOR*, mammalian target of rapamycin; *S6K1*, ribosomal protein S6 kinase; *4E-BP1*, eIF-4E binding protein-1; Ala, alanine; Gln, glutamine; LPS, lipopolysaccharide. Values are means ± SEM, *n* = 10. Different superscripts above the column denote differences, *p* < 0.05.

There were no interactions for *mTOR* and *S6K1* between LPS challenge and Gln supplementation (*p* > 0.05); however, a significant interaction for *4E-BP1* was observed between LPS challenge and Gln supplementation (*p* < 0.05). In comparison with the saline treatment, the LPS treatment significantly lowered the mRNA expressions of *mTOR*, 4*E-BP1*, and *S6K1* in the *Pectoralis Major* muscle of the broilers (*p* < 0.05, Figure 2). However, compared with the broilers fed the Ala diet, the mRNA expressions of both *mTOR* and *S6K1* in the broilers supplemented with Gln were elevated (*p* < 0.05). Moreover, diets supplemented with Gln reversed the reduction in the *4E-BP1* mRNA expression in the *Pectoralis Major* muscle of the broilers challenged with LPS stimuli (*p* < 0.05).

### 3.5. mRNA Expression of Akt/FOXO Signals Mediated by TLR4

As shown in Table 5, there were significant interactions for *TLR4*, *FOXO1*, and *MuRF1* between the LPS challenge and Gln supplementation (*p* < 0.05). However, no interactions for *Akt*, *FOXO4*, or *MAFbx* were observed between the LPS challenge and Gln supplementation (*p* > 0.05). When compared with broilers receiving saline treatment, LPS challenge significantly decreased *Akt* mRNA expression, but increased the mRNA expressions of *TLR4*, *FOXO4*, *FOXO1*, *MAFbx*, and *MuRF1* (*p* < 0.05). In contrast, compared with broilers fed the Ala diet, Gln supplementation upregulated the *Akt* mRNA expression and downregulated the mRNA expressions of *FOXO4* and *MAFbx* (*p* < 0.05). In addition, diets supplemented with Gln reversed the increased mRNA expressions of *TLR4*, *FOXO1*, and *MuRF1* in the *Pectoralis Major* muscle of the LPS-challenged broilers (*p* < 0.05).

## 4. Discussion

It has been demonstrated that LPS is a potential stimulant that triggers the release of proinflammatory cytokines [22,23]. The secretion of proinflammatory cytokines is crucial for activating the innate host defense system and subsequently regulating the adaptive immune response, such as IL-6, TNF-α, and IL-1β [24]. TNF-α, IL-6, and IL-1β, which originate from macrophages, are the major regulators of diverse inflammatory responses [5]. The results of our present study showed that LPS challenge increased the mRNA expressions of *TNF-α*, *IL-6*, and *IL-1β* in the liver, indicating that the LPS challenge induced an acute inflammatory response. It was demonstrated that the LPS challenge resulted in higher mRNA expressions of *TNF-α*, *IL-6*, and *IL-1β* in the liver of the broilers [25]. Similarly, increased mRNA expressions of *IL-6* and *IL-1β* were observed in the LPS-challenged broilers [24]. The results of our previous study suggested that Gln supplementation decreased the contents of TNF-α, IL-6, and IL-1β in the plasma of LPS-challenged broilers [5]. In addition, Gln, serving as a neutral and multifunctional essential amino acid, is particularly prominent in the antistress response [13,26]. Moreover, it was proven that Gln deprivation exacerbated the production of proinflammatory cytokines, whereas Gln supplementation limited the inflammatory response in vitro [27]. The results mentioned above indicate that Gln supplementation might help to alleviate the inflammatory responses induced by LPS challenge.

Because the physiological demand for Gln exceeds its synthesis capacity under catabolic stresses, it becomes a conditionally essential amino acid [28]. Skeletal muscle plays an important role in Gln metabolism and is quantitatively the most relevant site for Gln stock, synthesis, and release [29]. The two intracellular enzymes are GS and GA, which are responsible for Gln synthesis and Gln hydrolysis, respectively [30]. It has been proven that there is a concomitant increase in GA expression under catabolic conditions such as sepsis and infections [31,32]. Similarly, in our present study, LPS administration significantly increased the mRNA expression of *GA* in the muscles of the broilers, suggesting that the LPS challenge accelerated Gln consumption. In addition, an increased GS enzyme activity in skeletal muscle was observed during severe catabolic states [30]. In accordance with this, our results also showed that LPS challenge elevated the activity of GS in the muscle. Moreover, Gln metabolism is influenced by glutamine aminotransferase. In this study, Gln supplementation increased the AST activity in the muscle. Consistent with our results, it was also reported that 1% Gln supplementation increased the AST activity in the muscle, associated with Gln metabolism [33]. However, Gln addition significantly decreased *GA* mRNA expression in the muscle, indicating that Gln supplementation might have compensated for the decline in Gln in the muscle under stress and partly contributed to inhibiting the catabolism of Gln.

It has been well documented that the mTOR signaling pathway is an evolutionally conserved protein kinase and is important in regulating protein synthesis. The activation of mTOR and its downstream regulators 4E-BP1 and S6K1 synergistically leads to the initiation of polypeptide formation [34]. But, mTOR signaling is inhibited by sepsis and endotoxin-related inflammation [35]. In the present study, we found that LPS challenge downregulated the mRNA expressions of *mTOR*, *4E-BP1*, and *S6K1* in the muscle. It was also reported that the expressions of mTOR, 4E-BP1, and S6K1 were inhibited in septic rats or animals treated with LPS [35], indicating that protein synthesis was inhibited by LPS challenge. Independent of LPS administration, increased mRNA expressions of *mTOR*, combined with *4E-BP1* and *S6K1* in the muscle, were observed after Gln addition. In accordance with this, in our previous report, it was demonstrated that Gln supplementation ameliorated the growth performance of LPS-challenged broilers, evidenced by decreases in ADFI and ADG as well as the increase in F/G. It was reported that Gln addition increased the yield and weight of the breast muscle of broilers suffering from heat stress [36], indicating that Gln could contribute to ameliorating the development of breast muscle of broilers under stress conditions. In addition, it was demonstrated that Gln is required for the activation of mTOR signaling [37]. Similarly, in a previous study, Gln supplementation significantly elevated the protein expressions of mTOR, 4E-BP1, and S6K1 in skeletal muscle [28]. The addition of Gln to a medium stimulated protein synthesis through the mTOR signaling pathway [20]. The results mentioned above suggest that Gln contributes to promoting protein synthesis, associated with the activation of mTOR signaling.

The majority of intracellular proteins are degraded by the ubiquitin–proteasome pathway in all tissues [38], which contributes to 75% of protein degradation during skeletal muscle atrophy [39]. The activation of Akt and the inactivation of FOXO transcriptionally upregulated of the FOXO gene targets *MAFbx* and *MuRF1* and subsequently induced muscle protein degradation [40]. TLR4 was also demonstrated to be a master regulator of the muscle wasting induced by endotoxemia [41]. The present study found that LPS challenge significantly decreased the mRNA expression of *Akt* and increased the mRNA expressions of *TLR4*, *FOXO1*, *FOXO4*, *MAFbx*, and *MuRF1* in muscle. It was demonstrated that decreased Akt protein expression and increased mRNA expressions of *MAFbx* and *MuRF1* occurred in response to LPS-induced endotoxemia [42]. Furthermore, in a previous study, it was demonstrated that increased mRNA expressions of *FOXO1* and *FOXO4*, combined with increased *MAFbx* and *MuRF1* mRNA expressions, were induced by LPS challenge [7]. Similarly, in a previous study about LPS-administered rats, it was demonstrated that LPS challenge downregulated Akt expression and upregulated MAFbx and MuRF1 in skeletal muscle [40]. Currently, Gln was also shown to be an amino acid involved in the regulation of autophagy [43]. It was proven that Gln administration inhibited the protein degradation of intestinal epithelial cells [20]. Our results also showed that Gln addition increased the *Akt* mRNA expression and lowered the mRNA expression of *MAFbx* and *MuRF1*, which are associated with protein degradation. Moreover, in our previous study using piglets, alanyl-glutamine (a dipeptide of Gln) supplementation decreased mRNA expressions of both *MAFbx* and *MuRF1* in skeletal muscle under normal and LPS conditions [7,28]. In addition, Gln administration induced reduced mRNA expressions of *MAFbx* and *MuRF1*, and the loss in the skeletal muscle mass was alleviated partially by Gln supplementation [44]. Therefore, based on the results mentioned above, we speculate that Gln supplementation could contribute to inhibiting the protein degradation in skeletal muscle via the TLR4/Akt/UPP signaling pathway.

## 5. Conclusions

In summary, Gln supplementation alleviated inflammatory responses in the livers of LPS-challenged broilers. Moreover, Gln supplementation increased the expressions of the signaling molecules in the mTOR and Akt/FOXO/UPP pathways, indicating that Gln addition might contribute to promoting protein synthesis and inhibiting protein degradation of LPS-challenged broilers.

## Figures and Tables

**Table 1:** Ingredients and nutrient content of the basal diets.

Ingredients (g/kg Diet)		Nutrient Content (g/kg Diet)	
Maize	563.0	Crude protein ‡	210.8
Wheat bran	51.30	Metabolism energy (MJ/kg)	121.2
Soybean meal	285.0	Calcium (%)	10.00
Corn gluten meal	43.0	Phosphorus (%)	4.50
DL-methionine	1.50	DL-methionine (%)	8.60
Phytase	0.40	L-Lysine (%)	10.60
Choline	1.50	Threonine (%)	8.0
Dicalcium phosphate	18.70		
Limestone	12.60		
Salt	1.50		
Soybean oil	16.50		
Vitamin and mineral premix †	5.00		

† Premix per kg diet provided: vitamin A 12,000 IU; vitamin D3 2500 IU; vitamin E 30 mg; menadione 2.8 mg; thiamin 2.21 mg; riboflavin 7.8 mg; nicotinamide 40 mg; calcium pantothenate 10 mg; pyridoxine·HCl 4 mg; biotin 0.04 mg; folic acid 1.2 mg; Vitamin B12 0.015 mg; Fe 80 mg; Cu 8 mg; Mn 110 mg; Zn 65 mg; I 0.35 mg; Se 0.3 mg. ‡ Nutrient contents of the diets are the measured values.

**Table 2:** Primer sequences for RT-PCR analysis.

Genes	ID	Primer Sequences (5′-3′)	Product Size (bp)
*MAFbx*	NM_001030956.1	F: GCCAGTACCACTTCACAGACAGAC R: GCGTGTCACCATACTGCTCCTTC	132
*MuRF1*	XM_424369.4	F: GAACGACCGCATCCAGACCATC R: TCCGTCTTCTTCTCCTCCAGCAG	138
*FOXO1*	NM_204328.1	F: GACCTCATCACCAAGGCCATCG R: GCACGCTCTTGACCATCCACTA	85
*Akt*	NM_205055.1	F: GGCTACAAGGAACGACCGCAAG R: TACTGTGGTCCACTGGAGGCATC	141
*TLR4*	NM_001030693.1	F: TTCGGTTGGTGGACCTGAATCTTG R: ACAGCTTCTCAGCAGGCAATTCC	114
*GA*	NM_001031248.1	F: TCCTCGCAGAGAAGGTGGTGATC R: TACGTGCAATGCTGTTCGTGAGTC	154
*S6K1*	NM_001030721.1	F: GTTCAGGCTCACCCGTTCTTCAG R: TGGCTCACATCCTCTTCAGATTGC	107
*FOXO4*	XM_015278657.2	F: CAACGTTCCACCACCCGTGA R: TGGAGGCAGATTGCTGGGTA	101
*TNF-α*	NM_204267.1	F: TGTGTATGTGCAGCAACCCG R: AACAACCAGCTATGCACCCC	178
*mTOR*	XM_417614.6	F: AACCACTGCTCGCCACAATGC R: CATAGGATCGCCACACGGATTAGC	120
*4E-BP1*	XM_424384.6	F: GACCGTAAGTTCCTGATGGAGTGC R: ATTGGGCTGGTAACACCTGGAATG	92
*IL-1β*	NM_204524.1	F: AAGCCTCGCCTGGATTCTAG R: TCAGGTCGCTGTCAGCAAAG	90
*IL-6*	NM_204628.1	F: TCCCTCCTCGCCAATCTGAA R: AAATAGCGAACGGCCCTCAC	80
*β-actin*	NM_205518.1	F: ATTGTCCACCGCAAATGCTTC R: AAATAAAGCCATGCCAATCTCGTC	113

*MAFbx*, muscle atrophy F-box; *MuRF1*, muscle ring finger 1; *FOXO1*, forkhead box O1; *Akt*, protein kinase B, also named PKB; *TLR4*, toll-like receptor 4; *GA*, glutaminase; *S6K1*, ribosomal protein S6 kinase; *FOXO4*, forkhead box O4; *TNF-α*, tumor necrosis factor-α; *mTOR*, mammalian target of rapamycin; *4E-BP1*, eIF-4E binding protein-1; *IL-1β*: interleukin-1β; *IL-6*: interleukin-6.

**Table 3:** Effects of glutamine supplementation on the mRNA expressions of *TNF-α* (A), *IL-6* (B) and *IL-1β* (C) in the livers of LPS-challenged 21-day-old broilers.

Treatment	*TNF-α*	*IL-6*	*IL-1β*
Ala-saline	1.15	1.04 ^bc^	1.00 ^b^
Ala-LPS	1.87	1.48 ^a^	1.76 ^a^
Gln-saline	0.52	0.98 ^c^	0.69 ^c^
Gln-LPS	1.46	1.18 ^b^	1.03 ^b^
SEM	0.108	0.044	0.084
Main effect			
Diet			
Ala	1.51 ^a^	1.26 ^a^	1.38 ^a^
Gln	0.99 ^b^	1.08 ^b^	0.86 ^b^
Stress			
Saline	0.84 ^b^	1.01 ^b^	0.85 ^b^
LPS	1.67 ^a^	1.33 ^a^	1.40 ^a^
*p*-value			
Gln	<0.001	<0.001	<0.001
LPS	<0.001	<0.001	<0.001
Gln × LPS	0.117	0.005	<0.001

*TNF-α*, tumor necrosis factor-α; *IL-1β*: interleukin-1β; *IL-6*: interleukin-6; Ala, alanine; Gln, glutamine; LPS, lipopolysaccharide. Values are means ± SEM, *n* = 10. Different superscripts above the column denote differences, *p* < 0.05.

**Table 4:** Effects of Gln supplementation on the activities of ALT and AST in muscle of LPS-challenged 21-day-old broilers.

Treatment	ALT (u/g of Protein)	AST (u/g of Protein)
Ala-saline	2.93	22.97
Ala-LPS	2.43	16.49
Gln-saline	3.05	27.79
Gln-LPS	2.68	24.63
SEM	0.542	4.587
Main effect		
Diet		
Ala	2.68	19.73 ^b^
Gln	2.87	26.21 ^a^
Stress		
Saline	2.99	25.38 ^a^
LPS	2.56	20.56 ^b^
*p*-value		
Gln	0.603	<0.001
LPS	0.236	0.002
Gln × LPS	0.848	0.160

ALT, alanine aminotransferase; AST, aspartate aminotransferase; Ala, alanine; Gln, glutamine; LPS, lipopolysaccharide. Values are means and SEM, *n* = 10. Different superscripts in an array denote differences, *p* < 0.05.

**Table 5:** Effects of Gln supplementation on mRNA expressions of protein-degradation-related genes in the muscles of 21-day-old LPS-challenged broilers.

Treatment	*TLR4*	*Akt*	*FOXO4*	*FOXO1*	*MAFbx*	*MuRF1*
Ala-Saline	1.00 ^c^	1.00	0.99	1.04 ^b^	1.0	1.07 ^c^
Ala-LPS	1.43 ^a^	0.59	1.42	2.93 ^a^	1.4	2.81 ^a^
Gln-Saline	0.86 ^d^	1.39	0.92	0.94 ^b^	0.69	1.03 ^c^
Gln-LPS	1.01 ^b^	0.96	1.11	1.28 ^b^	1.14	1.47 ^b^
SEM	0.234	0.301	0.249	0.868	0.292	0.762
Main Effect						
Diet						
Ala	1.22 ^a^	0.80 ^b^	1.21 ^a^	1.99 ^a^	1.29 ^a^	1.94 ^a^
Gln	0.94 ^b^	1.18 ^a^	1.02 ^b^	1.11 ^b^	0.92 ^b^	1.25 ^b^
Stress						
Saline	0.93 ^b^	1.20 ^a^	0.96 ^b^	0.99 ^b^	0.89 ^b^	1.05 ^b^
LPS	1.22 ^a^	0.78 ^b^	1.27 ^a^	2.11 ^a^	1.32 ^a^	2.14 ^a^
*p* Value						
Gln	<0.001	<0.001	<0.001	<0.001	<0.001	<0.001
LPS	<0.001	<0.001	<0.001	<0.001	<0.001	<0.001
Gln × LPS	0.002	0.932	0.091	<0.001	0.266	<0.001

*TLR4*: toll-like receptor 4; *Akt*: protein kinase B; *FOXO4*: forkhead box O 4; *FOXO1*: forkhead box O 1; *MAFbx*: muscle atrophy F-box; *MuRF1*: muscle ring finger 1; Ala, alanine; Gln, glutamine; LPS, lipopolysaccharide. Values are means ± SEM, *n* = 10. Different superscripts in an array denote differences, *p* < 0.05.

## Data Availability

The data presented in this study are available on request from the corresponding author.
